# Human Chondrosarcoma Cells Acquire an Epithelial-Like Gene Expression Pattern via an Epigenetic Switch: Evidence for Mesenchymal-Epithelial Transition during Sarcomagenesis

**DOI:** 10.1155/2011/598218

**Published:** 2011-03-17

**Authors:** Matthew P. Fitzgerald, Francoise Gourronc, Melissa L. T. Teoh, Matthew J. Provenzano, Adam J. Case, James A. Martin, Frederick E. Domann

**Affiliations:** ^1^Free Radical and Radiation Biology Program, Department of Radiation Oncology, University of Iowa, Iowa City, IA 52242, USA; ^2^Department of Orthopaedics and Rehabilitation, University of Iowa, Iowa City, IA 52242, USA; ^3^Department of Otolaryngology-Head and Neck Surgery, University of Iowa, Iowa City, IA 52242, USA

## Abstract

Chondrocytes are mesenchymally derived cells that reportedly acquire some epithelial characteristics; however, whether this is a progression through a mesenchymal to epithelial transition (MET) during chondrosarcoma development is still a matter of investigation. We observed that chondrosarcoma cells acquired the expression of four epithelial markers, *E-cadherin,desmocollin 3, maspin*, and *14-3-3σ*, all of which are governed epigenetically through cytosine methylation. Indeed, loss of cytosine methylation was tightly associated with acquired expression of both *maspin* and *14-3-3σ* in chondrosarcomas. In contrast, chondrocyte cells were negative for *maspin* and *14-3-3σ* and displayed nearly complete DNA methylation. Robust activation of these genes was also observed in chondrocyte cells following 5-aza-dC treatment. We also examined the transcription factor *snail* which has been reported to be an important mediator of epithelial to mesenchymal transitions (EMTs). In chondrosarcoma cells *snail* is downregulated suggesting a role for loss of *snail* expression in lineage maintenance. Taken together, these results document an epigenetic switch associated with an MET-like phenomenon that accompanies chondrosarcoma progression.

## 1. Introduction

Chondrosarcoma is a rare but deadly form of bone cancer and is the second most common type of bone cancer accounting for nearly 26% of all bone cancers [1]. These tumors are stubbornly resistant to both chemotherapy and radiation therapy, therefore surgical ablation is still the most effective treatment [2, 3]. However since surgical resection is often difficult and not practical for metastatic disease, more effective treatments are needed. 

Chondrosarcomas have been presumed to arise from the chondrocyte lineage of mesenchymal cells; of mesodermal origin because they are the most similar cells however, the exact origin or subtype of cells is still an area of active investigation. Numerous studies have shown the occurrence of genetic alterations in chondrosarcomas including loss of heterozygosity (LOH) on multiple chromosomes, wide variation in ploidy status, and mutations in the tumor suppressors p53, p16ink4a, pRB, among others [4–6]. In contrast, relatively little is known about the epigenetic alterations that occur during chondrosarcoma progression [7, 8]. 

The malignant progression to chondrosarcoma has been suggested to involve some degree of a mesenchymal to epithelial transition (MET), and it has been shown in an *in vitro* model that chondrosarcoma cells can transition to a more epithelial-like phenotype under certain conditions [9]. MET is a fundamental developmental process which is important to vertebrate embryogenesis in vascular, urinary, and genital tissues [10, 11]. Although much has been learned about the more commonly known and well-studied reciprocal process, the epithelial to mesenchymal transition (EMT) during carcinoma progression, the mesenchymal to epithelial transition (MET) in sarcoma progression is considerably less well understood. These lineage transitions have important consequences to cell morphology, cell to cell adhesion, cell motility, and in the extracellular matrix of cells. However, the phenotypic plasticity conferred to cells as a result of these transitions which are so critical to development may also become coopted by cells during the process of carcinogenesis.

MET during carcinogenesis has been shown to be induced by the* c*-*met* proto-oncogene [9, 12, 13]. *P140 c*-*met *is a receptor tyrosine kinase for HGF/SF and increased expression leads to epithelial differentiation [14, 15]. In addition to epithelial specification by *c*-*met*, 5-azacytidine, a DNA methyltransferase inhibitor with broad spectrum epigenetic effects, has been used to induce the conversion of mesenchymal cells into epithelial cells *in vitro* [16]. More recently research on the transcription factor *snail* has been linked to aberrant DNA methylation of the epithelial specific E-cadherin promoter in association with EMT, and stable RNA interference of *snail* expression in carcinoma cell lines induced a complete MET [17, 18]. Finally, as corneal stromal keratinocytes differentiate into corneal fibroblasts they undergo an epigenetic switch with respect to *maspin* expression [19]. Such results highlight the possible role played by epigenetic changes through DNA methylation in a cell's ability to transdifferentiate from a mesenchymal to a more epithelial phenotype. 

To investigate whether chondrosarcoma cells are displaying some characteristics of MET we examined four epithelial markers to confirm the acquisition of more epithelial-like expression. These epithelial markers included *E*-*cadherin, desmocollin 3, maspin, and 14-3-3*σ*. *Next to investigate whether epigenetic changes are occurring in chondrosarcomas we examined protein and RNA expression along with the DNA methylation at two distinct and separate loci, *maspin* and *14-3-3*σ**. Both have been identified as specific epithelial markers and have separately been shown in lung, pancreas, prostate, and other cancers to be epigenetically controlled through DNA methylation [20–24]. Finally we measured expression of the *snail* transcription factor which has been reported to be an important mediator of EMT in part through epigenetic mechanisms [25].

*Maspin* is a member of the serpin family of protease inhibitors (*SERPINB5*) and has been described as an epithelial marker and a type II tumor suppressor gene based upon its ability to inhibit invasion and motility of mammary tumors [26–29]. Zhang and colleagues also found *maspin* to function as an inhibitor of angiogenesis [28, 30, 31]. *Maspin* gene expression is regulated in part through methylation of its promoter in human normal cells [29]. In addition, silencing of the *maspin* gene in association with aberrant DNA methylation has been reported in cancer cells from breast, melanoma, and thyroid [20, 32, 33]. Nevertheless, loss of *maspin* expression in cancer is not a universal phenomenon. In other malignancies such as pancreatic, lung, ovarian, and gastric cancers, *maspin* expression is paradoxically increased in malignant cells compared to their normal cells of origin [21, 34–36]. 

*14-3-3*σ**, also known as stratifin or HME1, was originally identified as an epithelial-specific marker downregulated in breast cancer cell lines [37]. *14-3-3*σ** has been shown to be involved in a wide variety of cellular processes, including its response to DNA damaging agents and gamma radiation through activation by p53, which then contributes to G2 cell cycle arrest [38, 39]. Studies have shown that, similar to *maspin*, downregulation of *14-3-3*σ** was associated with aberrant hypermethylation of the *14-3-3*σ** CpG island [23, 40, 41]. Since the original report, hypermethylation of *14-3-3*σ** leading to silencing has been reported in prostate, hepatocellular carcinomas, and others [23, 41]. However, just as with *maspin*, *14-3-3*σ** is not always downregulated and in fact is upregulated in pancreas and squamous cell carcinomas [42, 43]. 

Two members of the cadherin family of cell adhesion molecules *E-cadherin* and *desmocollin 3*, have been shown to be downregulated in several types of cancers through DNA methylation [44, 45]. This decrease in expression has been correlated with the epithelial to mesenchymal transition. The *snail* transcription factor has been shown to repress E-cadherin expression and has been reported to be an important mediator of epithelial to mesenchymal transitions. Recently, it has been shown that *snail* binds to the E-boxes of the E-cadherin promoter and can recruit the histone deacetylase HDAC1 and DNA methyltransferase DNMT1 to help in the epigenetic silencing of *E-cadherin * [46].

In this study we show an upregulation of four distinct epithelial markers and the downregulation of snail, all consistent with cells that have undergone to some extent an MET transition. Next we show that epigenetic alterations in two of these genes, *maspin* and *14-3-3*σ**, are consistent with their gain of expression in chondrosarcomas. We demonstrate that loss of DNA methylation at both the *maspin* and *14-3-3*σ** loci led to increased expression of these two epithelial specific genes during chondrosarcoma carcinogenesis. These results link the mesenchymal to epithelial transition in chondrosarcoma to an epigenetic switch in lineage-specific gene expression.

## 2. Materials and Methods

### 2.1. Cell Culture

Chondrosarcoma cells and normal chondrocytes were isolated by overnight digestion of chopped tissues with 0.5 mg/mL type IA collagenase and pronase E (Sigma) in Dulbecco Modified Eagle's medium with 10% fetal calf serum (Life Technologies). All cells were cultured as monolayers in growth medium containing 40% Dulbecco Modified Eagle's medium, 40% Minimum Essential medium, 20% Ham's F12, 10% fetal calf serum supplemented with 1.0 units/ml insulin, 20 *μ*g/mL hydrocortizone (Sigma), and 40 *μ*g/mL gentamycin or 100 units/ml penicillin/streptomycin and grown at 37°C with 5% CO_2_ in a humidified cell culture incubator. The SNM83 are the normal chondrocyte cell strain used in this study. The cell line JJ was a generous gift from Dr. Joel Block. The *in vitro* morphologies of several of these cell lines have been previously reported [47]. Briefly, we found that the *in situ* morphology of chondrocytes and low-grade chondrosarcoma cells changed in monolayer culture, where both transitioned from a spindle cell shape to a more polygonal cell shape after a few passages. High-grade (2-3) chondrosarcoma cells in monolayer culture retained their spindle shaped in culture.

### 2.2. Real-Time RT-PCR Assays for Gene Expression

Total cellular RNA was isolated using RNeasy Mini Kit (Qiagen, Valencia, CA, USA) and quantified using a biophotometer (Eppendorf, Westbury, NY, USA). For real-time RT-PCR analysis of *maspin* and *14-3-3*σ** mRNA expression, a reverse transcription step was performed using a High Capacity cDNA Archive Kit (Applied Biosystems Inc., Foster City, CA, USA). The reverse transcription reaction of 2 *μ*g of RNA was primed with random primers and incubated at 25°C for 10 min followed by 37°C for 120 min. The primer/probe PCR reactions consisted of 100 ng of cDNA added to 12.5 *μ*L of TaqMan Universal PCR Master Mix (Applied Biosystems Inc.), 1.25 *μ*L of gene-specific *maspin* and *14-3-3*σ** primer/probe mix (Assays-on-Demand, Applied Biosystems Inc.), and 6.25 *μ*L PCR grade water, for a 25 *μ*L total reaction. For *E-cadherin*, *desmocollin 3, *and *snail* the primers were designed using ABI primer express software. Primer sequences are available upon request. The PCR reactions consisted of 100 ng cDNA with 0.6 *μ*M primers in Power SYBR green PCR MasterMix (Applied Biosystems Inc.) with a total reaction volume of 25 *μ*L. The genes were not multiplexed but rather amplified in separate tubes. The PCR conditions for all reactions were 95°C for 10 min, followed by 40 cycles of 95°C for 15 s, with annealing at 60°C for 1 min. Real-Time PCR was performed on an ABI 7000 real-time sequence detection system. Both the *maspin* and *14-3-3*σ** gene-specific TaqMan probes were labeled with a 5′ reporter dye, 6-FAM, and a 3′ end containing a nonfluorescent quencher and a minor groove binder. Fold differences in mRNA expression were calculated using their respective mRNA expression calibrated to 18-s ribosomal RNA expression and computed using ABI relative quantitation software (Applied Biosystems Inc.).

### 2.3. Western Blot Analysis

Proteins were isolated from SNM83 and NH69 cells using RIPA buffer and quantified using a Bradford assay. Twenty *μ*g of protein were size fractionated by electrophoresis on a 12% SDS-PAGE gel and then transferred to nitrocellulose membranes. The membranes were then probed with a *maspin* antibody (Pharmingen) and *14-3-3*σ** antibody (Chemicon).

### 2.4. Sodium Bisulfite Genomic DNA (gDNA) Sequencing

Genomic DNA was isolated using the DNeasy Tissue Kit (Qiagen, Valencia, CA, USA) and quantified using a biophotometer (Eppendorf). Five micrograms of genomic DNA was modified under conditions previously described [32]. The *maspin* and *14-3-3*σ** CpG islands were amplified from the bisulfite modified DNA by two rounds of PCR utilizing nested PCR primers specific to the bisulfite modified sequence of the *maspin* promoter and the *14-3-3*σ** CpG island as described previously [32, 48]. The final PCR product was cloned into a TOPO TA vector according to the manufacturer's instructions (TOPO TA Cloning Kit, Invitrogen, Carlsbad, CA, USA). Five positive recombinants were isolated using Qiaprep Spin Plasmid Miniprep Kit (Qiagen) according to manufacturer's instructions and sequenced on an ABI automated DNA sequencer. The methylation status of individual CpG sites was determined by comparison of the sequence obtained with the known *maspin* and *14-3-3*σ** sequences. The number of methylated CpGs at a specific site was divided by the number of clones analyzed (*n* = 5) to yield the percent methylation for each site. For total promoter methylation calculation, the total of all the 19 CpG sites for *maspin* and the 27 CpG sites in *14-3-3*σ** that were methylated from the 5 clones was counted and divided by the total CpG sites.

### 2.5. Gene Reactivation Using 5-Aza-2′-Deoxycytidine

For 5-aza-2′-deoxycytidine (5-aza-dC) reactivation studies in the CS8E chondrosarcoma cell line, cells were plated at 5 × 104 cells in 6-well plates and were treated with 10 *μ*M 5-aza-dC in complete media on days 0, 2, and 4 then harvested for total RNA on day 5 using an RNeasy Mini Kit (Qiagen, Valencia, CA, USA). RNA was then analyzed by real-time RT-PCR.

## 3. Results

### 3.1. Gain of mRNA Expression of Epithelial-Specific Genes in Chondrosarcomas

**Figure 1:**
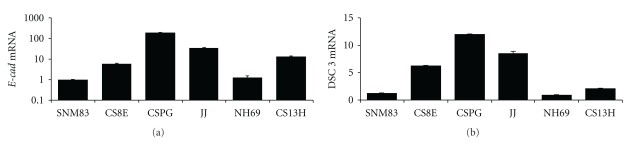
Chondrosarcomas acquire aberrant expression of epithelial-specific genes *E*-cadherin and desmocollin-3. (a) *E-cadherin* mRNA expression was measured by real-time PCR. Four of the five chondrosarcoma cell lines (CS8E, CSPG, JJ, and CS13H) showed a significant increase in *E-cadherin* mRNA expression from approximately 6- to 189-fold increase when compared to the normal SNM83 chondrocyte cell line. SNM83 had minimal expression but was set at 1 on graph as calculated using ΔΔCT relative quantification method. (b) *Desmocollin* 3 mRNA expression was measured by real-time PCR. Four of the five chondrosarcoma cell lines (CS8E, CSPG, JJ, and CS13H) showed a significant increase from approximately 1.5- to 12-fold more *Desmocollin* 3 mRNA when compared to normal line. Again SNM83 had minimal expression but was arbitrarily set at 1 for calculation purposes.

**Figure 2:**
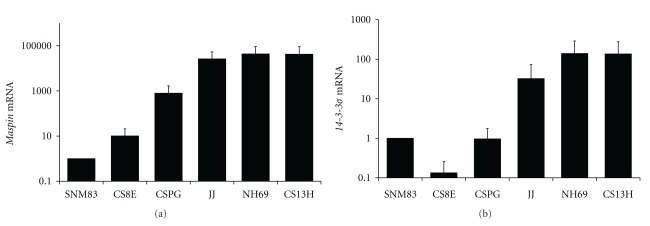
Chondrosarcomas acquire aberrant expression of epithelial-specific genes *maspin* and *14-3-3*σ**. (a) *Maspin* mRNA expression was measured by real-time PCR. Four of the five chondrosarcoma cell lines (CSPG, JJ, NH69, and CS13H) showed a significant increase in *maspin* mRNA expression when compared to the normal SNM83 chondrocyte cell line. SNM83 had minimal expression but was set at 1 on graph as calculated using ΔΔCT relative quantification method. (b) *14-3-3*σ** mRNA expression was measured by real-time PCR. Three of the five chondrosarcoma cell lines (JJ, NH69 and CS13H) showed a significant increase from approximately 50- to 150-fold more *14-3-3*σ** mRNA when compared to normal SNM83 and the CS8E and CSPG chondrosarcoma cell lines. Again SNM83 had minimal expression but was arbitrarily set at 1 for calculation purposes.

Compared to normal chondrocytes, chondrosarcoma cell lines displayed gain of expression of several epithelial-specific markers. *E-cadherin* mRNA expression was significantly upregulated in 4 of the 5 chondrosarcoma cell lines ranging from a 6- to 189-fold induction over the normal SNM83 normal chondrocyte cell strain as shown in Figure 1(a). Similarly, *desmocollin 3* mRNA expression was acquired in 4 of the 5 cell lines examined, ranging from a 2- to a 12-fold increase as compared to the normal counterpart SNM83, as shown in Figure 1(b). *Maspin* mRNA levels were also similarly affected. *Maspin* mRNA expression was virtually undetectable in the normal SNM83 chondrocyte cell strain and in stage I early CS8E chondrosarcoma cell line. In contrast, the chondrosarcoma cells CSPG, JJ, NH69, and CS13H displayed abundant *maspin* expression as shown in Figure 2(a) and Table 1. The chondrosarcoma cell lines displayed approximately 10^2^- to 10^5^-fold higher levels of *maspin* mRNA expression when compared to the normal SNM83 chondrocyte cell line. Finally, we determined that *14-3-3*σ** mRNA expression was also virtually undetectable in the SNM83 normal chondrocyte cell strain and in CS8E cancer cell line and low in the CSPG cell line. In the other chondrosarcoma cells JJ, NH69, and CS13H there was approximately 100-fold higher levels of *14-3-3*σ** mRNA expression as shown in Figure 2(b) and Table 1. To our knowledge this is the first report showing the upregulation of either *maspin* or *14-3-3*σ** in chondrosarcoma cell lines.

### 3.2. Gain of Epithelial-Specific Protein Expression

**Figure 3:**
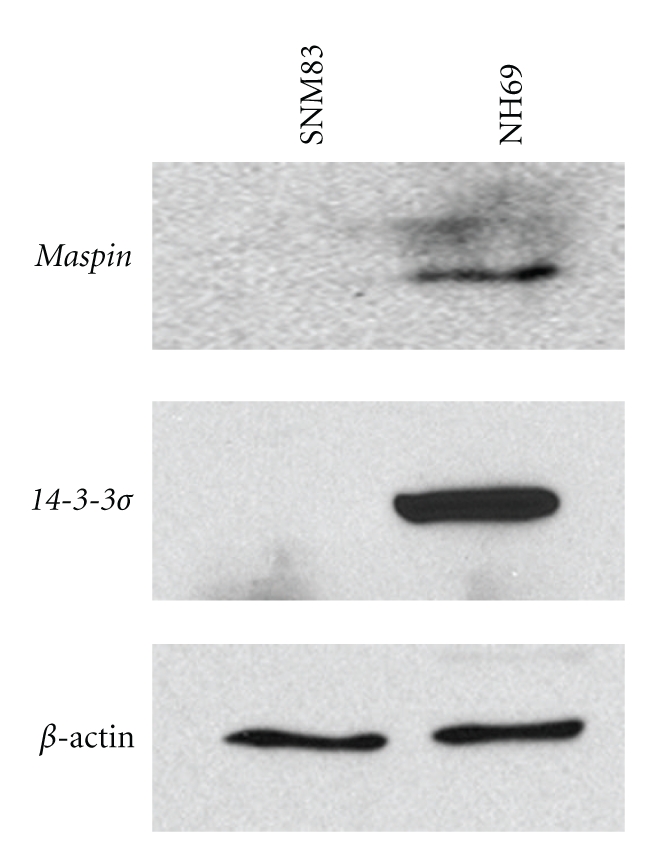
Chondrosarcoma cells expression of *maspin* and *14-3-3*σ** proteins. Western blot analysis for maspin and *14-3-3*σ**. Lane 1 is the SNM83 chondrocyte cell line which shows no protein expression for either maspin or *14-3-3*σ**, and in lane 2 the NH69 chondrosarcoma cell line shows a robust induction of both proteins. These are consistent with real-time PCR results of mRNA expression. Beta-actin was used as the loading control.

To determine whether the increases in mRNA observed were translated into functional proteins we performed western blotting on the epithelial-specific markers *maspin* and *14-3-3*σ** in two representative cell lines as shown in Figure 3. The normal SNM83 cells showed no detectable expression of *maspin* or *14-3-3*σ** while the chondrosarcoma cell line NH69 showed robust expression of both proteins consistent with the previously observed upregulation in the *maspin* and *14-3-3*σ**'s mRNA levels (Figure 2).

### 3.3. Maspin and 14-3-3*σ* Gene Methylation in Chondrocytes

**Figure 4:**
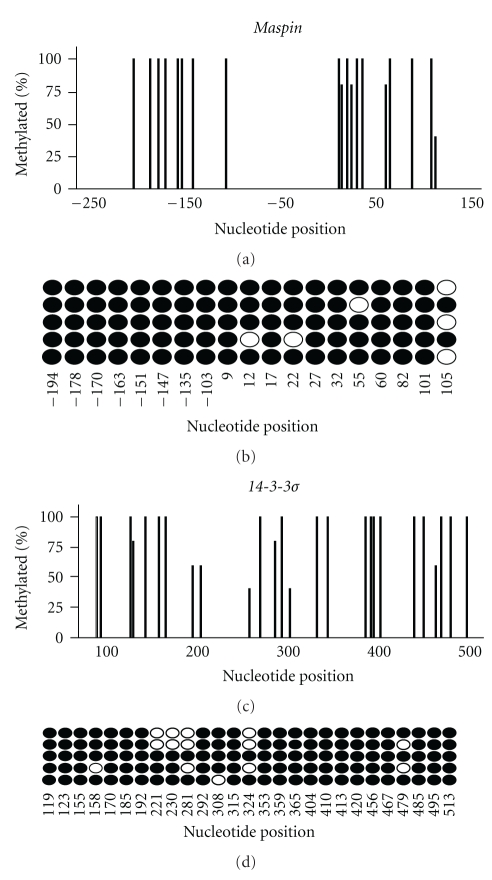
Normal chondrocyte cells have a highly methylated *maspin* promoter and *14-3-3*σ** CpG island. (a) and (c) Histograms representing the percent methylation at each of the CpG's spanning the *maspin* promoter and *14-3-3*σ** CpG island in SNM83 chondrocyte cells. (b) and (d) Bubble charts of Maspin and *14-3-3*σ** in SNM83. Each of the five rows represents a sequenced amplicon, while each of the columns represents the position of the nucleotide relative to the transcription start site. Darkened circles represent methylated cytosines while open circles represent unmethylated cytosines. Nucleotide positions relative to start site were based on UCSC genome browser build 17.

Figures 4(a) and 4(b) represent the normal methylated state of the *maspin* promoter in the normal chondrocyte cell line SNM83.  Figure 4(a) is a histogram representing the percent methylation at each of the 19 CpG locations and their distribution across the *maspin* promoter' whereas Figure 4(b) shows the five analyzed clones individually and the methylation at each CpG site. The overall methylation for the *maspin* promoter was 93% in the SNM83 cells (Table 1). This high degree of promoter methylation taken along with the undetectable SNM83 *maspin* mRNA and protein levels represented in Figures 2(a) and 3 is consistent with reported studies linking high *maspin* promoter methylation with silenced gene expression.

Figures 4(c) and 4(d) represent the methylation status of 27 CpG's in the *14-3-3*σ** gene in the SNM83 cells. Similar to the *maspin* promoter, *14-3-3*σ** shows a highly methylated state of the CpG's in normal *14-3-3*σ** negative chondrocytes.  Figure 4(c) is a histogram representing the overall percent methylation from the five picked clones at each CpG site and its distribution across the gene; whereas Figure 4(d) shows the individual clones and methylation at each CpG site. The overall methylation of SNM83 from the sequenced clones is 85% methylated. This methylation pattern of *14-3-3*σ**, similar to *maspin*, is associated with silenced *14-3-3*σ** expression.

### 3.4. Loss of Maspin Promoter Methylation in Human Chondrosarcomas

**Figure 5:**
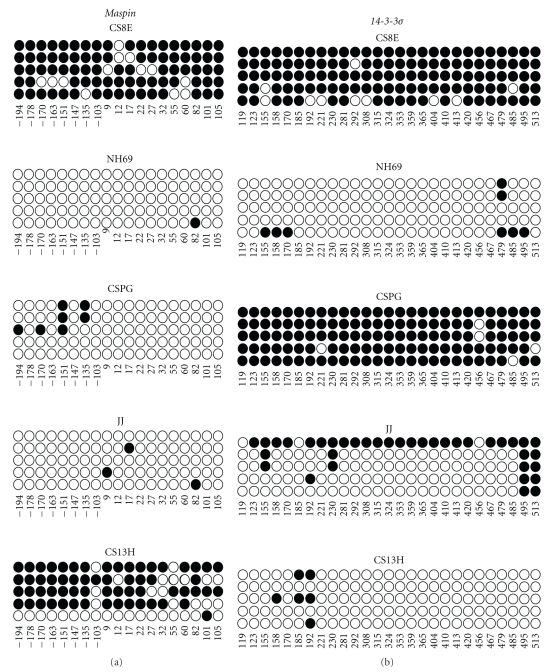
The *maspin* promoter and *14-3-3*σ** gene are hypomethylated in mRNA expressing chondrosarcoma cell lines. Bubble charts of the maspin promoter (a) and *14-3-3*σ** gene (b) for the five chondrosarcoma cell lines analyzed. Each of the five rows represents a sequenced amplicon while each column represents the nucleotide position of the CpG measured relative to the transcription start site. Darkened circles represent methylated cytosines, and clear circles represent unmethylated cytosines. Nucleotide positions relative to start site were based on UCSC genome browser build 17.

Figure 5(a) illustrates the methylation frequency and distribution at each of the 19 CpG's we measured in the *maspin* promoter in the five human chondrosarcoma cell lines analyzed. SNM83 (Figure 4) and CS8E displayed overall *maspin* promoter methylation percentages of 93% and 86%, respectively, while CS13H showed an intermediate methylation percentage of 65%. In sharp contrast, the remaining chondrosarcoma cell lines CSPG, JJ, and NH69 displayed significantly lower percentages of overall promoter methylation of 7%, 3%, and 1% respectively (Table 1). The intermediate promoter methylation of CS13H may be due to the phenotypic heterogeneity displayed in the tumor tissue from which the DNA was extracted (data not shown), or could be due to a reversion of this late stage chondrosarcoma back to a more “normal” methylation profile similar to chondrocytes. Nonetheless, these data are consistent with our previous report in human pancreatic carcinoma cell lines as well as reports from human breast, lung, ovarian, and thyroid cancers that tightly associate *maspin* expression to promoter methylation.

### 3.5. 14-3-3*σ* Gene Methylation in Chondrosarcomas

Figure 5(b) also illustrates the methylation frequency and distribution at each of the 27 CpG sites of the *14-3-3*σ** CpG island among the sequenced amplicons from the same five chondrosarcoma cell lines. CS8E and CSPG displayed a percentage of overall DNA methylation of 90% and 95%, respectively. In contrast, the remaining chondrosarcomas cell lines display relatively little DNA methylation (Table 1). This supports previous work with *14-3-3*σ** that shows the tight association of DNA methylation with low expression and hypomethylation with higher levels of *14-3-3*σ** expression [40, 42].

### 3.6. Gene Reactivation with 5-Aza-Deoxycytidine

**Figure 6:**
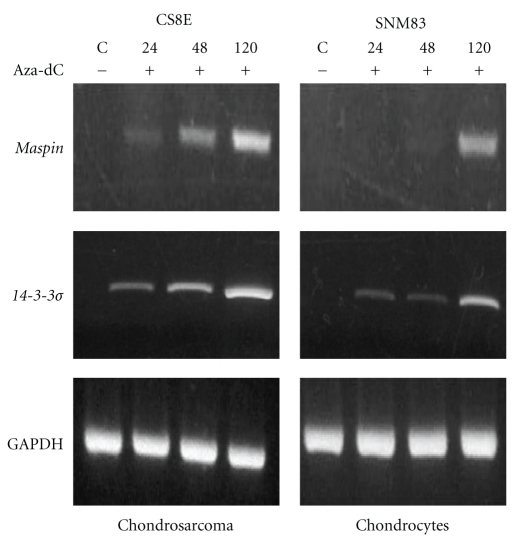
The methyltransferase inhibitor 5-Aza-dC reactivated both *maspin* and *14-3-3*σ** mRNA expression in chondrocytes and chondrosarcomas cell lines. The CS8E chondrosarcoma cell line and SNM83 normal chondrocyte cell line were exposed to 10 uM 5-Aza-dC. RNA was harvested after 48, 72, and 120 hours and subjected to RT-PCR for *maspin* and *14-3-3*σ**. GAPDH was used as loading control.

The mRNA expression and sodium bisulfite DNA sequencing data alone shows a potential association but does not fully establish a cause and effect relationship between cytosine methylation and *maspin* and *14-3-3*σ** gene expression. Therefore we investigated whether we could induce *maspin* and *14-3-3*σ** expression by treating the *maspin* and *14-3-3*σ** negative SNM83 chondrocyte cells and the CS8E chondrosarcoma cells with the DNA methyltransferase inhibitor 5-aza-2′-deoxycytidine (5-aza-dC). After 120 hours of 5 *μ*M 5-aza-dC both the SNM83 and CS8E cells showed a significant increase from almost undetectable levels of *maspin* and *14-3-3* *μ* to crisp mRNA expression when compared to the untreated controls as shown in Figure 6. These data are consistent with previous reports of induced *maspin* and *14-3-3*σ** expression following 5-aza-dC treatment of hypermethylated and nonexpressing cell lines [21, 32, 41, 49].

### 3.7. Loss of Snail mRNA Expression in Chondrosarcomas

**Figure 7:**
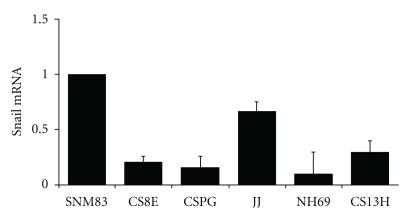
*Snail* mRNA expression is downregulated in human chondrosarcomas. All five chondrosarcoma cell lines showed a significant decrease ranging from 1.5- to a 10-fold decrease in *snail* mRNA expression when compared to the normal SNM83 chondrocyte cell line as determined by real-time PCR using ΔΔCT relative quantification method. Expression is normalized to SNM83 chondrocyte expression.

To begin to assess the potential underlying molecular mechanism(s) for the apparent epigenetic switch in lineage-specific gene expression we observed and we measured *snail* mRNA expression, since *snail* has been reported to participate in the MET. Interestingly, s*nail *mRNA expression was significantly downregulated in all five chondrosarcoma cell lines when compared to the SNM83 normal chondrocytes, ranging from a 1.5- to 10-fold decrease in mRNA expression as shown in Figure 7. This decrease is supportive of our observation that *E-cadherin *and other epithelial-specific markers mRNAs were induced in the majority of human chondrosarcoma cell lines analyzed and is noteworthy because of recent reports showing that stable RNA interference of *snail* can lead to a MET transition [25].

## 4. Discussion

In this study we examined whether epigenetic changes are associated with a mesenchymal to epithelial-like transition in chondrosarcomas. To first investigate whether that our chondrosarcoma cells were acquiring more epithelial-like characteristics we queried the expression of four separate epithelial markers: *E-cadherin, desmocollin 3, maspin,* and *14-3-3*σ**. All of these genes have been shown to be de-regulated in association with cytosine methylation and are involved with the malignant progression of many cancers [20–24, 50–52]. Therefore, two of these genes, *maspin and 14-3-3*σ**, were further examined as representatives for epigenetic alterations. We identified the acquisition of expression of all four epithelial-specific markers in four of the five chondrosarcoma cell lines examined. Moreover, the robust acquisition of *maspin* and *14-3-3*σ**expression in chondrosarcomas is associated with a significant loss of DNA methylation at those loci when compared to normal SNM83 chondrocyte cell strain.

The acquisition of the epithelial markers *E-cadherin* and *desmocollin 3* in four of the five chondrosarcoma cell lines is consistent with reports showing that sarcomas can to some degree transition through MET from their parental cell lineage [48, 53]. The acquisition of *E-cadherin* is interesting because of the numerous reports indicating that downregulation of *E-cadherin*, frequently by aberrant methylation, is a hallmark of EMT. An important mediator of EMT and *E-cadherin* downregulation in cancer is the zinc finger transcription factor *snail. *An example of *snail's* important role in EMT and control of *E-cadherin* has been shown in *snail* knock-out mice which show embryonic lethality, and the embryos fail to complete EMT, forming an altered mesodermal layer while still retaining *E-cadherin* expression. Among the chondrosarcoma cell lines assessed here, all five showed a significant decrease in the mRNA expression of the *snail* transcription factor. Although the amount of decrease in *snail* expression was not predictive of the fold mRNA increase of *E-cadherin*, the downregulation of *snail* is suggestive of a less repressed *E-cadherin* and therefore could help to explain the observed increase of *E-cadherin* in our chondrosarcoma cells. In recent reports *snail* has also been investigated as an effector of the epigenetic changes observed in the downregulation of *E-cadherin. *It has been shown in these reports that *snail* binds to the E-boxes of the *E-cadherin* promoter and helps to recruit both histone deacetylase 1 (HDAC1) and DNA methyltransferase 1 (DNMT1). Indeed when *snail* was stably overexpressed in Hep3b cells the *E-cadherin* promoter became hypermethylated and histone H3 and H4 acetylation were decreased [46]. In support of this, a similar study by Cano et al. showed that stable interference of *snail* mRNA in *snail* overexpressing Madin Darby canine kidney (MDCK) cells led to a full MET and re-expression of *E-cadherin*. They extended and confirmed these results when they stably knocked down *snail* in two mouse epidermal carcinoma cell lines with similar results [54]. 

The acquired expression in our chondrosarcoma cells of four epithelial markers that have been shown in other cancers to be deregulated by promoter methylation, taken together with the downregulation of *snail, *led us to examine whether epigenetic changes could be associated with this MET-like transition in the chondrosarcoma cell lines assessed. To do this we compared the methylation status of *maspin* and *14-3-3*σ** in chondrosarcoma cells that acquired the expression of these markers to their nonexpressing normal counterpart, the SNM83 chondrocyte cell line. We report here that acquisition of both *maspin* and *14-3-3*σ** in chondrosarcoma cell lines is tightly associated with aberrant hypomethylation of their CpG islands and that expression of both epithelial markers could be induced in a nonexpressing chondrosarcoma cell line by addition of the DNA methyltransferase inhibitor 5-Aza-dC. These findings are consistent with the epigenetic control of these loci documented in recent studies of multiple normal and cancer cell types [21, 22, 34, 55]. The acquisition of *maspin* and *14-3-3*σ** in chondrosarcomas and other cancers, while still seemingly paradoxical to its role as a tumor suppressor, may be considered as a loss of epigenetic control. However, this may be better viewed as an epigenetic switch whereby the cancer cell, during progression, coopts the normal epigenetic mechanism(s) to propagate diverse cell types of differing lineage specificities. An example of this epigenetic switch may occur in solid tumors such as in colorectal cancer which uses the EMT transition to acquire a more metastatic phenotype but subsequently undergoes the reciprocal MET at the site of metastasis to reacquire, at least in part, the phenotype of the originating tumor [10]. This phenotypic reversion may confer a selective advantage to its new environment at the site of metastasis and thus allow for more successful colonization. The dynamic interconversions between EMT and MET in malignant progression cannot simply be explained by irreversible genetic alterations. These interconversions are suggestive of epigenetic mechanisms playing a role in these transitions because epigenetic changes, in contrast to irreversible genetic changes, are not permanent and allow a tumor cell more plasticity to alter its gene expression to adapt to different environments that can ultimately lead to phenotypic changes. 

One of the ways a tumor cell can accomplish this epigenetic switch is through variable DNA methylation of the CpG sites in the transcriptional control regions of genes. Specific examples of this switch in *maspin* and *14-3-3*σ** expression have been documented to occur both in ovarian and breast carcinomas. It is interesting to note that *maspin* expression is silenced during breast cancer progression, but activated during ovarian cancer progression [34, 35]. Ovarian surface epithelial cells being derived from the mesoderm activate *maspin* expression through DNA hypomethylation as they transition through MET [56]. Conversely, breast carcinomas typically silence *maspin* expression through hypermethylation and undergo the EMT to become more mesenchymal [32, 40, 57]. It is also noteworthy to mention that *snail* expression has been shown to be decreased in ovarian cancer cells during MET and to increase in breast cancer cells during EMT [58, 59]. To speculate that *snail* is affecting the epigenetic control of *maspin* expression in these cancers as well as in our chondrosarcoma cells is intriguing but has yet to be examined. However a bioinformatics search of both *maspin* and *14-3-3*σ** promoters reveals putative *snail* binding sites but further research is needed to elucidate any direct interaction. These examples may however help to explain the paradoxical gain of expression of *maspin* and *14-3-3*σ** in chondrosarcomas as well as in pancreatic and ovarian cancers. While epigenetic changes in the expression of *maspin*, *14-3-3*σ**, *desmocollin 3*, and *E-cadherin* in chondrosarcoma cells and in other cancers are associated with the EMT or MET transitions, the extent to which they are a contributing factor to these process remains to be determined. Nevertheless, they may provide new biomarkers for differential diagnosis of cartilaginous diseases and provide better insights into how cells loss lineage maintenance in cancer. 

In addition to showing an epigenetic mechanism for *14-3-3*σ** upregulation in chondrosarcoma cells it is interesting to speculate on what affects this acquired expression could have on treatment. The abundant expression of *14-3-3*σ** might help to confer the drug and radiation resistance commonly found in the treatment of chondrosarcomas [4, 40, 60]. In a recent study *14-3-3*σ** was identified as an important contributor to drug resistance in human breast and pancreas cancer cells, and the exogenous overexpression of *14-3-3*σ** led to a greater resistance to chemotherapeutics and radiation [60, 61].

## 5. Conclusion

In summary we show that chondrosarcoma cells acquire four epithelial-specific markers *maspin, 14-3-3*σ*, desmocollin 3*, and *E-cadherin,* which when taken together, is suggestive of chondrosarcoma cells undergoing to some degree an MET transition. We also report that all of the chondrosarcomas examined showed a significant downregulation of the *snail *transcription factor which may help to explain the re-acquisition of *E-cadherin* and MET-like transition in our cell lines. The reports that *snail* has been shown to act as an epigenetic repressor of *E-cadherin* by recruitment of histone deacetylase 1 and DNA methyltransferase led us to examine whether additional changes in the epigenetic maintenance of two well-known epithelial markers *maspin* and *14-3-3*σ** were occurring during this MET-like transition. We show here for the first time that chondrosarcoma cells acquire both *maspin *and *14-3-3*σ** mRNA expression which is associated with vastly decreased DNA methylation of their genes. The acquisition of expression of these genes could be playing a role in malignant progression, or their expression could simply be biomarkers of progression. Expression of these genes, especially 14-3-3*σ*, could also be contributing to some of the characteristics of chondosarcomas such as resistance to chemotherapy and radiation [40]. We now report that epigenetic changes through loss of DNA methylation occur to activate epithelial specific genes *maspin* and *14-3-3*σ** and that they are associated with the upregulation of *E-cadherin, desmocollin3* and the downregulation of *snail* during the transition of chondrocytes to chondrosarcomas. These epigenetic changes have not been extensively studied in MET, and this new knowledge could lead to more insight into the mechanisms underlying this important process, as well as aid in identifying new markers for better staging, diagnosing, and treating chondrosarcomas.

## Figures and Tables

**Table 1:** Summary of *maspin* and 14-3-3*σ* mRNA expression and cytosine methylation states in human chondrosarcoma cell lines.

Cell Line	Expression	Fold change mRNA	Methylation	% Methylation
*Maspin*				

SNM83	−	1	+	93
CS8E	−	10	+	86
CSPG	+	1000	−	7
JJ	+	32,000	−	3
NH69	+	63,000	−	1
CS13H	+	63,000	+/−	65

*14-3-3*σ**				

SNM83	−	1	+	85
CS8E	−	.15	+	90
CSPG	−	1	+	95
JJ	+	45	−	27
NH69	+	150	−	6
CS13H	+	145	−	4.4

## Abbreviations

5-Aza-dC: 5-Aza-2′-deoxycytidine
LOH: Loss of heterozygosity
MET: Mesenchymal to Epithelial transition
EMT: Epithelial to Mesenchymal transition
E-cad: E-Cadherin
DSC3: Desmocollin 3.
